# Chemical Constituents of *Phaius mishmensis*

**DOI:** 10.3390/molecules21111605

**Published:** 2016-11-23

**Authors:** Chen-Wei Jao, Tzu-Heng Hung, Chi-Fen Chang, Ta-Hsien Chuang

**Affiliations:** 1 Department of Chemistry, National Cheng Kung University, Tainan 701, Taiwan; foxrain.tw@msa.hinet.net; 2 School of Pharmacy, China Medical University, Taichung 40402, Taiwan; u103003093@cmu.edu.tw; 3 Department of Anatomy, School of Medicine, China Medical University, Taichung 40402, Taiwan; 4 Research Center for Chinese Herbal Medicine, China Medical University, Taichung 40402, Taiwan

**Keywords:** *Phaius mishmensis*, Orchidaceae, phaindole, (7′*R*,8′*R*)-phaithrene, methyl 3-hydroxy-4,5-dimethoxypropiophenone, methyl hematinate

## Abstract

The partitioned *n*-hexane, CHCl_3_, and EtOAc extracts from the crude MeOH extract of *Phaius mishmensis* showed considerable cytotoxicities against the human breast carcinoma (MCF-7), lung carcinoma (NCI-H460), and central nervous system carcinoma (SF-268) cell lines. Four new compounds, phaindole (**1**), (7′*R*,8′*R*)-phaithrene (**2**), methyl 3-hydroxy-4,5-dimethoxypropiophenone (**3**), and methyl hematinate (**4**), as well as 44 known compounds were isolated from the MeOH extract of *Phaius mishmensis*. The structures of the compounds were determined using spectroscopic methods.

## 1. Introduction

In our search for novel anticancer compounds from Taiwanese plants, the crude MeOH extract of *Phaius mishmensis* (Orchidaceae), a native orchid of Taiwan [1], showed considerable cytotoxicity against the human breast carcinoma (MCF-7), lung carcinoma (NCI-H460), and central nervous system carcinoma (SF-268) cell lines. Thus, the plant *P. mishmensis* was selected for purification based on the preliminary results. We isolated eight indoloquinazolinones, phaitanthrins A–E, methylisatoid, tryptanthrin, and candidine from the CHCl_3_-soluble extract of the plant [2]. After extensive column and preparative thin-layer chromatographic separations, four new compounds, phaindole (**1**), (7′*R*,8′*R*)-phaithrene (**2**), 3-hydroxy-4,5-dimethoxypropiophenone (**3**), and methyl hematinate (**4**), as well as 44 known compounds were isolated from the MeOH extract of *P. mishmensis*. Herein we describe the isolation, substance elucidation, and cytotoxic properties of the isolated compounds.

## 2. Results and Discussion

### 2.1. Isolation of Chemical Constituents of P. mishmensis

**Figure 1:**
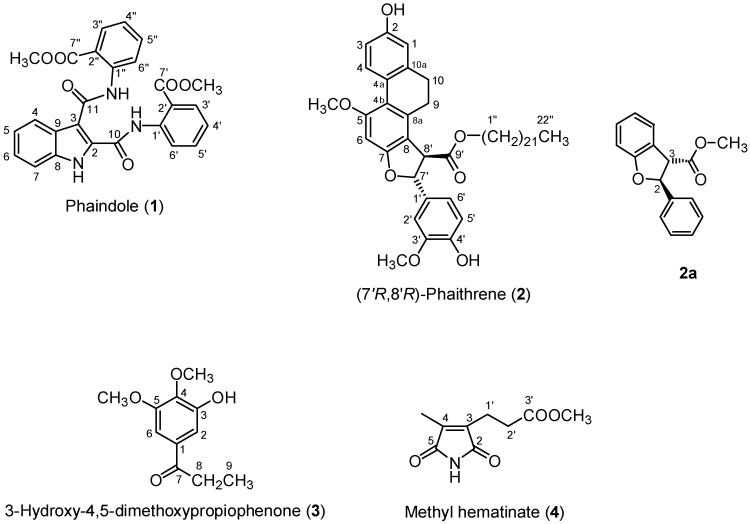
Structures of (2*S*,3*S*)-methyl 2,3-dihydro-2-phenylbenzofuran-3-carboxylate (**2a**) and four new compounds **1**–**4**.

Compound **1**, isolated as a yellowish powder, was determined to have the molecular formula C_26_H_22_N_3_O_6_ as noted by the pseudo-molecular ion peak at *m*/*z* 472.1506 in high resolution electrospray ionization mass spectroscopy (HR-ESIMS). A broad infrared (IR) absorption at 3264 cm^−1^ indicated the presence of a hydroxyl or amino functionality. Furthermore, four strong IR absorptions at 1702, 1697, 1659, and 1650 cm^−1^ might indicate the presence of two carbonyl and two amidic functionalities. The ^1^H-NMR (500 MHz) and ^13^C-NMR (125 MHz) spectral data of compound **1** were shown in Table 1, and all chemical shifts (δ) were given in ppm. In the aromatic region of the ^1^H- and COSY spectra, three sets of four mutually coupled proton signals at δ 7.34 ppm (t, *J* = 8.0 Hz, H-5), 7.40 ppm (t, *J* = 8.0 Hz, H-6), 7.52 ppm (d, *J* = 8.0 Hz, H-7), 8.14 ppm (d, *J* = 8.0 Hz, H-4), 7.22 ppm (t, *J* = 8.0 Hz, H-4′), 7.56 ppm (t, *J* = 8.0 Hz, H-5′), 7.98 ppm (d, *J* = 8.0 Hz, H-3′), 8.14 ppm (d, *J* = 8.0 Hz, H-6′), 7.16 ppm (t, *J* = 8.0 Hz, H-4′′), 7.62 ppm (t, *J* = 8.0 Hz, H-5′′), 8.07 ppm (d, *J* = 8.0 Hz, H-3′′), and 8.86 ppm (d, *J* = 8.0 Hz, H-6′′) were observed. Combined with ^13^C-, HMQC, and HMBC data, we determined that the first *o*-disubstituted benzene was fused with a pyrrole ring to form an indole unit, as noted by the HMBC correlations of H-1 (δ 9.98 ppm, br s, NH) with C-3 (δ 112.3 ppm), C-7 (δ 112.5 ppm), and C-9 (δ 125.7 ppm), and H-4 with C-3, whereas the latter two signals belonged to two methyl 2-aminobenzoate moieties, as determined by the HMBC correlations of H-3′ and 7′-OCH_3_ (δ 3.83 ppm) with C-7′ (δ 167.1 ppm), H-3′′ and 7′′-OCH_3_ (δ 3.85 ppm) with C-7′′ (δ 168.4 ppm), as well as 1′-NH (δ 12.55 ppm) with C-6′ (δ 124.8 ppm) and 1′′-NH (δ 11.85 ppm) with C-2′′ (δ 116.0 ppm) and C-6′′ (δ 121.6 ppm). The remaining two carbonyl signals at δ 159.2 ppm (C-10) and 164.7 ppm (C-11) were attributed to C-2 and C-3 of the indole ring, respectively, which formed an amidic linkage with two methyl 2-aminobenzoate groups as determined by the HMBC correlations of 1′-NH with δ 159.2 ppm (C-10) and 1′′-NH with δ 164.7 ppm (C-11). The NOE correlations between H-1 and H-7, 1′-NH and H-6′, 1′′-NH and H-4, and H-6′′ revealed that the structure of **1** was 2,3-di(2-methoxycarbonylphenyl)carbamoylindole; this compound was named phaindole (Figure 1).

The optically active compound **2** ([α]_D_ −18.5°) was obtained as a colorless powder and determined to have the molecular formula C_47_H_67_O_7_, owing to the pseudo-molecular ion peak at *m*/*z* 743.4883 in HR-FABMS. The IR spectrum showed a broad absorption at 3399 cm^−1^ corresponding to a hydroxyl group and a strong absorption at 1728 cm^−1^ corresponding to a carbonyl group. The ^1^H-NMR (300 MHz) and ^13^C-NMR (75 MHz) spectral data of compound **2** were shown in Table 1. In the aromatic region of the ^1^H-NMR and COSY spectra, three mutually coupled proton signals at δ 6.69 ppm (d, *J* = 2.6 Hz, H-1), 6.71 ppm (dd, *J* = 8.2, 2.6 Hz, H-3), and 8.09 ppm (d, *J* = 8.2 Hz, H-4) corresponding to a trisubstituted benzene ring and singlet proton signal at δ 6.53 ppm (s, H-6) for a pentasubstituted benzene ring were observed. In the aliphatic region of the ^1^H-NMR spectrum, an ethylene proton signal at δ 2.66 ppm (4H, m, H-9, and H-10) was observed. Using HMQC, HMBC, and NOESY, key long-range ^1^H-^13^C correlations of H-1 with C-10 (δ 29.7 ppm); H-4 with C-4b (δ 116.8 ppm); H-9 with C-4b , C-8 (δ 113.9 ppm), C-10a (δ 139.3 ppm); H-10 with C-1 (δ 114.1 ppm) and C-8a (δ 136.8 ppm), as well as a key NOE correlation between H-1 and H-10 revealed a 2,5,7,8-tetrasubstituted 9,10-dihydrophenanthrene nucleus. Three very down-field–shifted ^13^C- signals at C-2 (δ 153.4 ppm), C-5 (δ 158.5 ppm), and C-7 (δ 159.0 ppm) together with the NOE correlations between a hydroxyl group (δ 4.76 ppm) and H-1, -3, a methoxyl group (δ 3.87 ppm) and H-4, -6, revealed a hydroxyl (2-OH), a methoxyl (5-OCH_3_), and oxygenated substituents (7-OR). The remaining three unresolved aromatic proton signals at δ 6.88 ppm (3H, m, H-2′, H-5′, and H-6′), a hydroxyl signal at δ 5.63 ppm (4′-OH), a methoxyl signal at δ 3.88 ppm (3′-OCH_3_), -CHCH- proton signals at δ 4.21 ppm (d, *J* = 5.4 Hz, H-8′) and 5.93 ppm (d, *J* = 5.4 Hz, H-7′), together with a carbonyl signal at δ 172.4 ppm (C-9'), revealed a 7′-oxygenated-8′-alkyl dihydroferulate moiety. The HMBC correlations of 4′-OH with C-3′ (δ 146.7 ppm) and C-5′ (δ 114.5 ppm); 3′-OCH_3_ with C-3′ (δ 146.7 ppm); H-7′ with C-2′ (δ 108.0 ppm), C-6′ (δ 118.6 ppm), and C-9′; H-8′ with C-1′ (δ 132.8 ppm) and C-9 confirmed the presence of a ferulate moiety. A 22-long chain alcohol at δ 0.88 ppm (3H, t, *J* = 6.8 Hz, H-22″), 1.25 ppm (38H, m, H-3″–H-21″), 1.62 ppm (2H, quintet, *J* = 6.9 Hz, H-2″), and 4.15 ppm (2H, t, *J* = 6.9 Hz, H-1″) formed docosyl ferulate, as noted by the HMBC correlation of H-1′′ with C-9′. Finally, the HMBC correlation of H-7′ with C-7, -8; H-8′ with C-7; and the NOE correlation between H-8′ and H-9 revealed that the ferulate was fused to the phenanthrene ring to form a furanophenanthrene. Based on a report by Juhasz [3], the absolute configuration was determined as follows: first, the smaller coupling constant 5.5 Hz between H-7′ and H-8′ suggested that the substituents on the dihydrofuran ring were in the *trans* configuration. Second, a similar compound, (2*S*,3*S*)-methyl 2,3-dihydro-2-phenylbenzofuran-3-carboxylate (**2a**), with the *S* configuration at C-2 showed a negative Cotton effect at 280 nm by circular dichroism (CD) spectrometry. Compound **2** presented a positive Cotton effect at 280 nm by CD spectrometry, indicating the 7′*R* configuration. Consequently, the absolute configuration of (7′*R*,8′*R*) was deduced for **2** and the compound was named (7′*R*,8′*R*)-phaithrene.

Compound **3** was isolated as yellowish needles, and was determined to have the molecular formula C_11_H_14_O_4_ by HR-EIMS at *m*/*z* 210.0894. A broad IR absorption at 3390 cm^−1^ and a strong IR absorption at 1680 cm^−1^ revealed the existence of hydroxyl and carbonyl groups, respectively. The ^1^H-NMR (300 MHz) and ^13^C-NMR (75 MHz) spectral data of compound **3** are shown in Table 1. The only two ^1^H-NMR signals at δ 7.16 ppm (d, *J* = 1.9 Hz, H-6) and 7.22 ppm (d, *J* = 1.9 Hz, H-2) indicated a 1,3,4,5-tetrasubstituted benzene nucleus. Three oxygenated substituents, namely a hydroxyl group at δ 5.87 ppm and two methoxyl groups at δ 3.91 ppm and 3.97 ppm, were observed. The fourth substituent was determined to be a propanoyl group due to an ethyl signal at δ 1.21 ppm (3H, t, H-9) and 2.94 ppm (2H, q, H-8) with a coupling constant of 7.2 Hz, which showed HMBC correlations with a carbonyl carbon at δ 199.8 ppm (C-7). The attachments were confirmed by the HMBC correlations between H-2, -6, and C-7, indicating that the propanoyl group was present on C-1; the HMBC correlations between the hydroxyl group at δ 5.87 ppm and H-2 indicated that the hydroxyl group was on C-3. Two methoxyl groups were present on C-4 and -5. Finally, the NOE correlation between a methoxyl group at δ 3.91 ppm (5-OCH_3_) and H-6 and a methylene group at δ 2.94 ppm (H-8) and H-2, -6 confirmed that the structure of **3** was 3-hydroxy-4,5-dimethoxypropiophenone.

Compound **4** was isolated as a white solid and was confirmed to have the molecular formula C_9_H_12_NO_4_ by the HR-FABMS signal at *m*/*z* 198.0767. From the ^1^H NMR spectrum (Table 1), the structure of **4** was determined to possess a mutually coupled ethylene group at δ 2.61 ppm (H-2′) and 2.71 ppm (H-1′), a methyl group at δ 2.00 ppm (4-CH_3_), and a methoxyl group at δ 3.67 ppm (3′-OCH_3_). The HMBC correlation of the 3′-OCH_3_ with a carbonyl C-3′ (δ 172.5 ppm); H-1′, H-2′, and 4-CH_3_ with accidently coinciding olefinic carbons C-3 and C-4 (δ 139.7 ppm); as well as a NOE correlation between 4-CH_3_ and H-1′ indicated a *cis* CH_3_C=CCH_2_CH_2_COOCH_3_ fragment. The remaining two carbonyl carbon signals at δ 171.4 ppm (C-2) and 171.5 ppm (C-5) and a broad NH proton signal at δ 7.52 ppm as well as the HMBC correlations between H-1′ and C-2 and 4-CH_3_ and C-5 formed an imido -O=CNHC=O- fragment. By combining these two fragments, the structure of 3-(methoxycarbonyl)ethyl-4-methyl-2,5-pyrroledione was thus deduced as methyl hematinate (**4**). This compound has been obtained from the photooxygenation of biliverdin [4]. However, this is the first time it has been isolated naturally.

**Figure 2:**
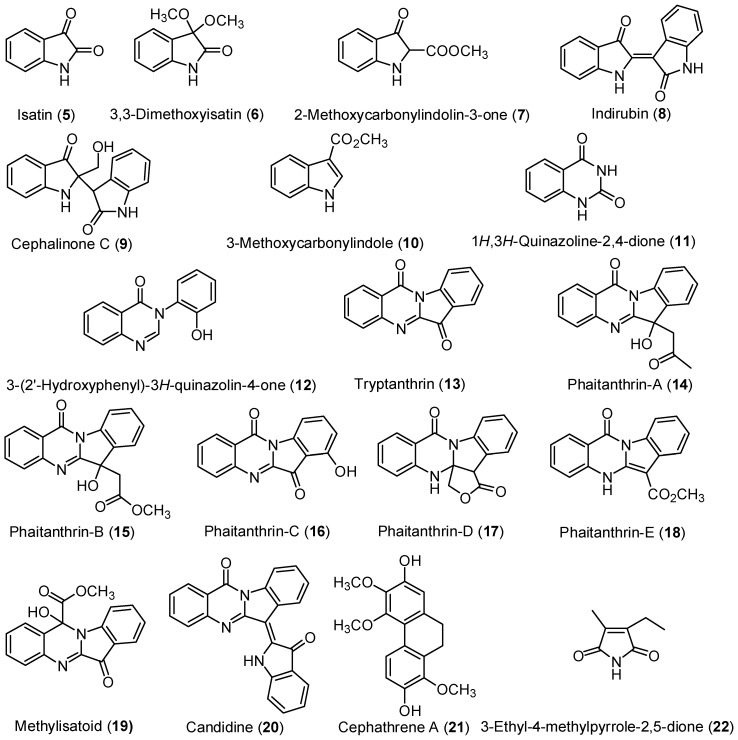
Structures of these known compounds **5**–**22**.

Other known compounds were also isolated from *P. mishmensis*, including 44 known compounds. The compounds were six indoles: isatin (**5**) [5], 3,3-dimethoxyisatin (**6**) [6], 2-methoxycarbonylindolin-3-one (**7**) [7], indirubin (**8**) [8], cephalinone C (**9**) [9,10], 3-methoxycarbonylindole (**10**) [11]; 10 quinazolines: 1*H*,3*H*-quinazoline-2,4-dione (**11**) [12], 3-(2'-hydroxyphenyl)-3*H*-quinazolin-4-one (**12**) [13], tryptanthrin (**13**) [2], phaitanthrin-A (**14**) [2], phaitanthrin-B (**15**) [2], phaitanthrin-C (**16**) [2], phaitanthrin-D (**17**) [2], phaitanthrin-E (**18**) [2], methylisatoid (**19**) [2], candidine (**20**) [2]; one phenanthrene: cephathrene A (**21**) [10]; one imide: 3-ethyl-4-methylpyrrole-2,5-dione (**22**) [14] (Figure 2).

**Figure 3:**
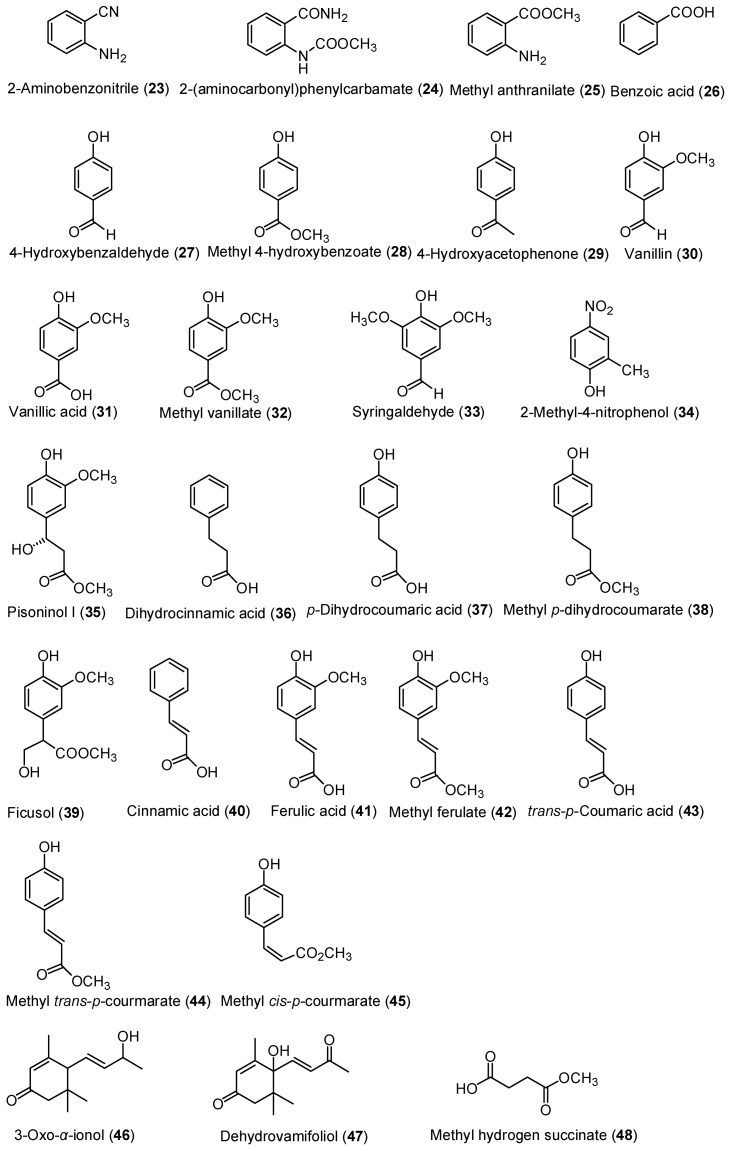
Structures of monocyclic aromatic hydrocarbons **23**–**45** and compounds **46**–**48**.

Moreover, 23 monocyclic aromatic hydrocarbons, 2-aminobenzonitrile (**23**) [15], 2-(aminocarbonyl)phenylcarbamate (**24**) [10], methyl anthranilate (**25**) [16], benzoic acid (**26**) [17], 4-hydroxybenzaldehyde (**27**) [18], methyl 4-hydroxybenzoate (**28**) [18], 4-hydroxyacetophenone (**29**) [18], vanillin (**30**) [10], vanillic acid (**31**) [10], methyl vanillate (**32**) [17], syringaldehyde (**33**) [10], 2-methyl-4-nitrophenol (**34**) [19], pisoninol I (**35**) [20], dihydrocinnamic acid (**36**) [21], *p*-dihydrocoumaric acid (**37**) [10], methyl *p*-dihydrocoumarate (**38**) [22], ficusol (**39**) [17], cinnamic acid (**40**) [18], ferulic acid (**41**) [10], methyl ferulate (**42**) [10], *trans*-*p*-coumaric acid (**43**) [23], methyl *trans*-*p*-courmarate (**44**) [18], and methyl *cis*-*p*-courmarate (**45**) [24], as well as 3-oxo-α-ionol (**46**) [25], dehydrovamifoliol (**47**) [26], and methyl hydrogen succinate (**48**) [27] were also isolated (Figure 3).

### 2.2. Cytotoxicity of Chemical Constituents of P. mishmensis

The partitioned *n*-hexane, CHCl_3_, and EtOAc extracts from the crude MeOH extract of *P. mishmensis* showed considerable cytotoxicities against the MCF-7, NCI-H460, and SF-268 cell lines (Table 2). Unfortunately, most of the isolated compounds, except tryptanthrin (**13**) and phaitanthrin-A (**14**) [28], did not exhibit significant cytotoxicity against the tested cell lines. This result suggested that the practically insoluble tryptanthrin (**13**) could disperse in organic layers during extraction.

## 3. Materials and Methods

### 3.1. General

Optical rotations were measured on a Jasco DIP-370 digital polarimeter (JASCO, Tokyo, Japan). CD spectra were determined on a Jasco J-715 spectropolarimeter (JASCO, Tokyo, Japan). UV spectra were recorded on an Agilent 8453 spectrophotometer (Agilent Technologies, Palo Alto, CA, USA). The IR spectra measured using a Nicolet Magna FT-IR spectrophotometer (Nicolet Instrument, Inc., Madison, WI, USA). The ^1^H-, ^13^C-, and 2D NMR spectra were recorded on Bruker Avance 300, AMX 400, and Avance-500 FT-NMR spectrometers (Bruker, Karlsruhe, Germany) at room temperature. All chemical shifts (δ) are given in ppm using tetramethylsilane as an internal standard. Mass spectra were obtained on a VG 70-250S spectrometer by a direct inlet system (Micromass Corp., Manchester, UK).

### 3.2. Plant Material

Whole plants of *P. mishmensis* were collected from Nanto Hsien, Taiwan, in October 2003. The collection was authenticated by Professor Chang-Sheng Kuoh, Department of Life Sciences, National Cheng Kung University, Tainan, Taiwan. A voucher specimen (No. PLW-0304) was deposited in the Herbarium of National Cheng Kung University, Tainan, Taiwan.

### 3.3. Extraction and Isolation

The air-dried *P. mishmensis* plants (3.5 kg) were extracted with MeOH (7 × 8 L) under reflux. The combined extracts were concentrated under reduced pressure to give a dark brown syrup. The syrup was suspended in H_2_O and then partitioned successively with *n*-hexane, CHCl_3_, and EtOAc. These concentrated layers were stored in a refrigerator at −20 °C before they were purified.

The concentrated *n*-hexane layer (81 g) was fractionated on a silica gel column by eluting with a gradient of *n*-hexane and Me_2_CO (9:1, *v*/*v* to 100% Me_2_CO) to obtain eight fractions. Fractions 1–5 were included fatty acids, chlorophylls, sitosterol, and stigmasterol. Fraction 6 was chromatographed on a silica gel column with *n*-hexane–EtOAc (4:1, *v*/*v*) to obtain 3-methoxycarbonylindole (**10**) (3.9 mg), phaindole (**1**) (3.4 mg), tryptanthrin (**13**) (total 350 mg), cephathrene A (**21**) (2.4 mg), (7′*R*,8′*R*)-phaithrene (**2**) (18.1 mg), 3-hydroxy-4,5-dimethoxypropiophenone (**3**) (4.8 mg), and methyl ferulate (**42**) (6.8 mg).

The concentrated CHCl_3_ layer (15 g) was chromatographed on a silica gel column by eluting with a gradient of CHCl_3_ and MeOH (20:1, *v*/*v* to 100% MeOH) to yield seven fractions. After repeated chromatography on silica gel followed by preparative TLC, fraction 2 gave 2-methoxycarbonylindolin-3-one (**7**) (3.0 mg), tryptanthrin (**13**) (total 350 mg), phaitanthrin-A (**14**) (30.0 mg), candidine (**20**) (9.8 mg), 2-aminobenzonitrile (**23**) (2.9 mg), methyl anthranilate (**25**) (3.1 mg), and methyl vanillate (**32**) (1.2 mg). Fraction 3 yielded isatin (**5**) (2.7 mg), 3,3-dimethoxyisatin (**6**) (1.5 mg), tryptanthrin (**13**) (total 350 mg), methylisatoid (**19**) (1.8 mg), phaitanthrin-D (**17**) (11.8 mg), phaitanthrin-E (**18**) (2.0 mg), 3-ethyl-4-methylpyrrole-2,5-dione (**22**) (1.0 mg), methyl hematinate (**4**) (8.6 mg), 4-hydroxybenzaldehyde (**27**) (11.1 mg), methyl 4-hydroxybenzoate (**28**) (7.8 mg), 4-hydroxyacetophenone (**29**) (4.2 mg), vanillin (**30**) (31.3 mg), syringaldehyde (**33**) (12.2 mg), 2-methyl-4-nitrophenol (**34**) (0.5 mg), and methyl *p*-dihydrocoumarate (**38**) (54.4 mg) by eluting with *n*-hexane and Me_2_CO (4:1, *v*/*v* to 100% Me_2_CO). Fraction 4 was chromatographed on a silica gel column by eluting with a gradient of CHCl_3_ and MeOH (50:1 to 20:1, *v*/*v*) to yield 3-(2′-hydroxyphenyl)-3*H*-quinazolin-4-one (**12**) (57.0 mg), tryptanthrin (**13**) (total 350 mg), phaitanthrin-C (**16**) (3.1 mg), 2-(aminocarbonyl)phenylcarbamate (**24**) (285.3 mg), benzoic acid (**26**) (1.3 mg), pisoninol I (**35**) (5.2 mg), dihydrocinnamic acid (**36**) (6.1 mg), ficusol (**39**) (9.0 mg), cinnamic acid (**40**) (1.2 mg), 3-oxo-α-ionol (**46**) (2.4 mg), and dehydrovamifoliol (**47**) (2.5 mg). Fraction 5 was chromatographed on a silica gel column by eluting with a gradient of CHCl_3_ and MeOH (50:1 to 4:1, *v*/*v*) to obtain indirubin (**8**) (36.8 mg), cephalinone C (**9**) (19.8 mg), tryptanthrin (**13**) (total 350 mg), phaitanthrin-B (**15**) (3.6 mg), methyl *trans*-*p*-courmarate (**44**) (0.7 mg), and methyl *cis*-*p*-courmarate (**45**) (0.5 mg).

The concentrated EtOAc layer (17 g) was chromatographed on a silica gel column by eluting with a gradient of CHCl_3_ and MeOH (10:1, *v*/*v*) and 2% H_2_O to yield six fractions. Fraction 2 was chromatographed on a silica gel column by eluting with a gradient of CHCl_3_ and MeOH (50:1, *v*/*v*) to give tryptanthrin (**13**) (total 350 mg). Fraction 2 was separated on a silica gel column by eluting with a gradient of CHCl_3_ and MeOH (50:1, *v*/*v*) to obtain tryptanthrin (**13**) (total 350 mg), vanillic acid (**31**) (55.3 mg), and methyl hydrogen succinate (**48**) (261.1 mg). Fraction 3 was chromatographed on a silica gel column by eluting with a gradient of CHCl_3_ and MeOH (50:1, *v*/*v*) to yield *p*-dihydrocoumaric acid (**37**) (8.4 mg), *trans*-*p*-coumaric acid (**43**) (1.6 mg), and ferulic acid (**41**) (0.7 mg). In addition, a solid (345.5 mg) insoluble in CHCl_3_ and MeOH was identified as 1*H*,3*H*-quinazoline-2,4-dione (**11**).

*Phaindole* (**1**): yellowish powder, UV (CHCl_3_) λ_max_ (log ε) 254 (3.89), 320 (3.90), 412 (2.68) nm; IR (KBr) ν_max_ 3264, 1702, 1697, 1659, 1650, 1605 cm^−1^; EIMS *m/z* (rel. int.) 471 (2, [M]^+^), 86 (100); HR-ESIMS *m/z* 472.1506 [M + H]^+^ (calcd for C_26_H_22_N_3_O_6_, 472.1506).

*(7'R,8'R)-Phaithrene* (**2**): colorless amorphous powders; [α]_D_ − 18.5° (*c* 0.08, CHCl_3_); UV (CHCl_3_) λ_max_ (log ε) 240 (3.94), 282 (4.01) nm; IR (KBr) ν_max_ 3399, 1728, 1609 cm^−1^; FABMS *m/z* (rel. int.) 743 (25, [M + H]^+^), 389 (100); HR-FABMS *m/z* 743.4883 [M + H]^+^ (calcd for C_47_H_67_O_7_, 743.4886).

*3-Hydroxy-4,5-dimethoxypropiophenone* (**3**): colorless needles, UV (CHCl_3_) λ_max_ (log ε) 238 (3.47), 274 (3.82) nm; IR (KBr) ν_max_ 3390, 1680 cm^−1^; EIMS *m/z* (rel. int.) 210 (49, [M]^+^), 181 (100); HR-EIMS *m/z* 210.0894 [M]^+^ (calcd for C_11_H_14_O_4_, 210.0892).

*Methyl hematinate* (**4**): white powder; UV (MeOH) λ_max_ (log ε) 230 (3.80), 270 (3.06) nm; IR (film) ν_max_ 3295, 2955, 1776, 1731, 1714 cm^−1^; FABMS *m/z* (rel. int.) 198 ([M + H]^+^, 22), 149 (100); HR-FABMS *m/z* 198.0767 [M + H]^+^ (calcd for C_9_H_12_NO_4_, 198.0766).

### 3.4. Cytotoxicity Assay

The cytotoxicity assay was carried out according to a procedure described previously [29]. Carcinoma cells MCF-7 and SF-268 were maintained in DMEM (Dulbecco’s Modified Eagle Medium, Fisher Scientific, HyClone, Logan, UT, USA) and NCI-H460 were maintained in RPMI (Roswell Park Memorial Institute, MP Biomedicals, Inc., Solon, OH, USA) medium supplemented with 10% fetal bovine serum (Biological Industries Inc., Cromwell, CT, USA). Firstly, the MCF-7, NCI-H460, and SF-268 cells were plated at a density of 5 × 10^3^ cells per well in 96-well plates overnight and then treated with different concentrations of the isolated compounds. After 48 h, MTS cell proliferation assay kit (Promega, Madison, WI, USA) was added to each well; then, the experiment was performed as the manufacturer recommended (Promega). The absorbance was measured at 490 nm on a MQX200R microplate reader (BioTek, Winooski, VT, USA).

## 4. Conclusions

Four new compounds, phaindole (**1**), (7′*R*,8′*R*)-phaithrene (**2**), methyl 3-hydroxy-4,5-dimethoxypropiophenone (**3**), and methyl hematinate (**4**), and 44 known compounds were isolated from *P. mishmensis*. Most of the isolated compounds, except tryptanthrin (**13**) and phaitanthrin-A (**14**), did not exhibit any significant cytotoxicity against the MCF-7, NCI-H460, and SF-268 cell lines. Phaitanthrin-A (**14**), an aldol adduct of tryptanthrin with acetone, exhibited better solubility than compound **13** in commonly used solvents (e.g., chloroform, ethyl acetate, and methanol). Derivatives using tryptanthrin as a template are being produced in our laboratories to obtain tryptanthrin derivatives with better solubilities and stronger tumor-selective toxicities.

## Figures and Tables

**Table 1:** ^13^C- and ^1^H-NMR spectroscopic data for **1**–**4**.

Position	1 in CDCl_3_ (125 MHz/500 MHz)		2 in CDCl_3_ (75 MHz/300 MHz)		3 in CDCl_3_ (75 MHz/300 MHz)		4 in CDCl_3_ (75 MHz/300 MHz)	
	δ_C_ ppm	δ_H_ ppm (*J* in Hz)	δ_C_ ppm	δ_H_ ppm (*J* in Hz)	δ_C_ ppm	δ_H_ ppm (*J* in Hz)	δ_C_ ppm	δ_H_ ppm (*J* in Hz)
1		9.98 br s	114.1	6.69 d (2.6)	132.6			7.52 br s
2	133.4		153.4		108.8	7.22 d (1.9)	171.4	
3	112.3		112.8	6.71 dd (8.2, 2.6)	148.9		139.7	
4	121.4	8.14 d (8.0)	129.2	8.09 d (8.2)	139.4		139.7	
4a			125.8					
4b			116.8					
5	122.5	7.34 t (8.0)	158.5		152.2		171.5	
6	125.5	7.40 t (8.0)	92.7	6.53 s	103.7	7.16 d (1.9)		
7	112.5	7.52 d (8.0)	159.0		199.8			
8	134.4		113.9		31.6	2.94 q (7.2)		
8a			136.8					
9	125.7		27.0	2.66 m	8.4	1.21 t (7.2)		
10	159.2		29.7	2.66 m				
10a			139.3					
11	164.7							
1′	137.9		132.8				19.3	2.71 m
2′	122.0		108.0	6.88 m			31.7	2.61 m
3′	130.9	7.98 d (8.0)	146.7				172.5	
4′	124.6	7.22 t (8.0)	145.8					
5′	133.0	7.56 t (8.0)	114.5	6.88 m				
6′	124.8	8.14 d (8.0)	118.6	6.88 m				
7′	167.1		87.4	5.93 d (5.4)				
8′			56.0	4.21 d (5.4)				
9′			172.4					
1″	141.2		65.7	4.15 t (6.9)				
2″	116.0		28.6	1.62 quintet (6.9)				
3″	131.0	8.07 d (8.0)	25.8	1.25 m				
4″	123.1	7.16 t (8.0)	29.5					
5″	134.4	7.62 t (8.0)						
6″	121.6	8.86 d (8.0)						
7″	168.4							
8″–19″								
20″			31.9					
21″			22.7					
22″			14.1	0.88 t (6.8)				
2-OH				4.76 br s				
3-OH						5.87 br s		
4-CH_3_							8.7	2.00 s
4-OCH_3_					61.0	3.97 s		
5-OCH_3_			55.6	3.87 s *	56.0	3.91 s		
1′-NH		12.55 s						
3′-OCH_3_			55.8	3.88 s *			51.9	3.67 s
4′-OH				5.63 br s				
7′-OCH_3_	52.2	3.83 s						
1″-NH		11.85 s						
7″-OCH_3_	52.5	3.85 s						

* Assignments may be interchangeable.

**Table 2:** Percentage inhibition of four partitioned extracts from the MeOH extract of *P. mishmensis* toward three cancer cell lines.

Extracts	% Inhibition at 20 μg/mL		
	MCF-7 ^1^	NCI-H460 ^2^	SF-268 ^3^
*n*-hexane	3	1	25
CHCl_3_	2	1	4
EtOAc	1	1	1
H_2_O	134	90	114

^1^ MCF-7 = human breast tumor cell line. ^2^ NCI-H460 = human lung tumor cell line. ^3^ SF-268 = human central nervous system tumor cell line.
